# Statement of Retraction: Neuroprotective effects of ellagic acid on cuprizone-induced acute demyelination through limitation of microgliosis, adjustment of CXCL12/IL-17/IL-11 axis and restriction of mature oligodendrocytes apoptosis

**DOI:** 10.1080/13880209.2024.2374677

**Published:** 2024-07-04

We, the Editors and Publisher of the journal *Pharmaceutical Biology*, have retracted the following article:

Nima Sanadgol, Fereshteh Golab, Zakiyeh Tashakkor, Nooshin Taki, Samira Moradi Kouchi, Ali Mostafaie, Mehdi Mehdizadeh, Mohammad Abdollahi, Ghorban Taghizadeh & Mohammad Sharifzadeh (2017) Neuroprotective effects of ellagic acid on cuprizone-induced acute demyelination through limitation of microgliosis, adjustment of CXCL12/IL-17/IL-11 axis and restriction of mature oligodendrocytes apoptosis, *Pharmaceutical Biology,* 55:1, 1679-1687, DOI: 10.1080/13880209.2017.1319867

Since publication, significant concerns have been raised about the fact that this article has substantial article content overlap with the following article:

Sanadgol N, Golab F, Mostafaie A, Mehdizadeh M, Abdollahi M, Sharifzadeh M, Ravan H. Ellagic acid ameliorates cuprizone-induced acute CNS inflammation via restriction of microgliosis and down-regulation of CCL2 and CCL3 pro-inflammatory chemokines. *Cell Mol Biol* (Noisy-le-grand). 2016 Oct 31;62(12):24-30. doi: 10.14715/cmb/2016.62.12.5.

This overlap is present in the form of text similarities reported in the methods, results and conclusions of the article which has not been appropriately cited or acknowledged.

Upon investigation, further data integrity concerns were identified; this included Figure 3B of the article. Figure 3B has been reused from Figure 1 of Sanadgol et al. (2016) however, this figure represents different experimental timescales.

As verifying the validity of published work is core to the integrity of the scholarly record, we are therefore retracting the article. All authors listed in this publication have been informed. The authors agree with the retraction.

We have been informed in our decision-making by our policy on publishing ethics and integrity and the COPE guidelines on retractions.

The retracted article will remain online to maintain the scholarly record, but it will be digitally watermarked on each page as “Retracted”.

